# Persistent *Helicobacter pylori* Specific Th17 Responses in Patients with Past *H. pylori* Infection Are Associated with Elevated Gastric Mucosal IL-1β

**DOI:** 10.1371/journal.pone.0039199

**Published:** 2012-06-25

**Authors:** Victoria Serelli-Lee, Khoon Lin Ling, Cassandra Ho, Lai Han Yeong, Gek Keow Lim, Bow Ho, Soon Boon Justin Wong

**Affiliations:** 1 Department of Microbiology, National University of Singapore, Singapore, Republic of Singapore; 2 Department of Gastroenterology and Hepatology, Singapore General Hospital, Singapore, Republic of Singapore; 3 Immunology Programme, National University of Singapore, Singapore, Republic of Singapore; Vanderbilt University Medical Center, United States of America

## Abstract

**Background:**

Ongoing *Helicobacter pylori* (HP) infection triggers a chronic active gastritis. Eradicating HP reduces gastric inflammation, but does not eliminate it. We sought to characterize this persistent gastritis, and demonstrate the persistence of HP-specific Th17 responses in individuals previously infected with HP but who no longer had evidence of ongoing infection.

**Methodology/Principal Findings:**

Study subjects were divided into 3 groups 55 individuals had active HP infection (group A), 41 were diagnosed with previous HP infection (group P), and 59 were naïve to HP (group N). Blood and gastric tissue were obtained with written informed consent from all subjects, and immune responses were evaluated using flow cytometry, semi-quantitative real time PCR, immunofluorescent staining, ELISA, and multiplex cytometric bead array for cytokine quantification. Elevated IL-17A responses were observed in patients from group A compared to group N. Interestingly, IL-17A responses remained persistently elevated in the blood and gastric mucosa of individuals from group P, despite the absence of ongoing HP infection. Using purified CD4^+^ T cells as effectors and antibodies that blocked antigen presentation by MHC Class II, we showed that these persistent IL-17A responses were mediated primarily by HP-specific Th17 cells, rather than other immune cells that have also been described to secrete IL-17A. Gastric mucosal IL-1β levels were also persistently elevated in group P, and neutralisation of IL-1β reduced the HP-specific IL-17A response of purified CD4^+^ T cells to autologous HP-pulsed antigen presenting cells in vitro, suggesting a functional association between IL-1β and the persistent Th17 response in group P patients.

**Conclusions/Significance:**

Despite lack of ongoing HP infection, HP-specific Th17 cells persist in the blood and gastric mucosa of individuals with past HP infection. We speculate that this persistent inflammation might contribute to gastric mucosal pathology, for example, persistent increased gastric cancer risk despite eradication of HP.

## Introduction

*Helicobacter pylori* (HP) infects the human stomach, and has been associated with various gastric diseases, including gastritis, peptic ulcer disease, gastric adenocarcinoma, and gastric mucosa-associated lymphoma. [1] Infection of the gastric epithelium is sensed by Toll-like receptors and NOD-like receptors, and triggers an inflammatory response characterized by elevated levels of pro-inflammatory cytokines, e.g. IL-1β, IL-6, IL-8, IL-18, TNF-α, and the recruitment of neutrophils and lymphocytes into the gastric mucosa. [2], [3], [4] Nevertheless, HP evades this vigorous response to establish a persistent infection that co-exists with chronic active inflammation of the gastric mucosa. [3].

Gastric mucosal lymphocytes isolated from patients infected with HP contain increased numbers of CD4^+^ T cells that produce IFNγ, consistent with prominent Th1 polarization. [5], [6], [7] More recently, ongoing HP infection has also been associated with upregulation of IL-17A expression in the gastric mucosa. [8], [9], [10] IL-17A is the most widely studied member of the IL-17 family of cytokines (IL-17A – F), and is produced by Th17 CD4^+^ T cells as well as other subsets of immune cells. [11], [12] Extracellular bacterial and fungal infections elicit strong IL-17A responses that stimulate stromal and epithelial cells to release pro-inflammatory cytokines and chemokines, e.g. TNF-α, IL-1β, IL-6, CXCL1, CXCL2, CCL2, CCL7, CCL20, which recruit neutrophils, macrophages and lymphocytes to the site of infection. [13], [14] IL-17A also induces expression of matrix metalloproteinases 1, 2, 3, 9, and 13, which regulate inflammation by modulating chemokine activity and establishing chemotactic gradients. [15] On the other hand, pathological persistence of IL-17A responses has been associated with tissue damage in the setting of chronic inflammatory and autoimmune diseases. [12], [14] IL-17A has also been implicated in the pathogenesis of various cancers, [16], [17], [18] including gastric cancer, [19], [20] although the biological basis of this association remains unclear.

Even though HP eradication is now possible with the use of antimicrobial agents, [21] significant lymphocytic infiltrate can remain in the gastric mucosa more than a decade following successful treatment of HP infection. [22] However, this chronic lymphocytic infiltrate has not been further characterized. Since chronic IL-17A signaling has pathological associations, we wanted to determine whether IL-17A responses contribute to persistent gastric inflammation after HP eradication, and the types of immune cells that produced IL-17A under these conditions.

Previous studies identified upregulated IL-17A expression during HP infection by comparing individuals with ongoing HP infection versus “HP negative” (uninfected, or naive) individuals. [8], [10] In this study, the association between IL-17A and HP infection was re-evaluated by also including patients with past HP infection in the comparison. These were individuals without evidence of ongoing HP infection or gastric cancer, but who either had a past history of treatment for HP infection or tested seropositive for HP. By studying such individuals, persistent HP-specific Th17 responses were detected in the blood and gastric mucosa despite absence of ongoing HP infection. These responses were associated with elevated IL-1β levels in the gastric mucosa.

**Table 1:** Patient data.

	All	Active HP infection(Group A)	Past HP infection (Group P)	Naïve (Group N)
*n*	155	55	41	59
Mean age in years ± SD	55±12	56±11	59±9	51±13
Numbers of male subjects (% of malesubjects in the respective groups)	70 (46%)	34 (62%)	18 (44%)	19 (32%)

## Materials and Methods

### Patient Samples and Classification

This research was conducted according to the principles expressed in the Declaration of Helsinki, and was approved by the Centralized Institutional Review Board (CIRB), Singapore Health Services, Singapore. Peripheral blood and gastric biopsy samples were obtained with written informed consent from 155 patients who underwent gastroscopy at the Singapore General Hospital (patient data summarized in table 1). Patients were asked to stop proton pump inhibitors and antibiotics 2 weeks and 4 weeks prior to endoscopy respectively. For each patient, biopsies were obtained from the gastric antrum, incisura, body, and cardia. Six of these biopsy samples were sent for histological assessment with hematoxylin and eosin stain, one for rapid urease test, and two samples were cultured for HP. Patients were classified as having ‘Active’ infections (Group A) if HP was identified in histology, rapid urease test, or culture. Patients without evidence of active HP infection but who either had a past history of treatment for HP infection, or who tested seropositive for HP, were classified as having ‘Past’ infection (Group P). [23] The HP serological status was determined by an immunoblot assay, Helicoblot 2.1 (MP Biomedicals, Singapore). ‘Naive’ patients (Group N) had no evidence of either an active or past HP infection. Pre-cancerous lesions were defined as the presence of either chronic atrophic gastritis or intestinal metaplasia in gastric biopsy samples that had been sent for histological assessment.

**Table 2:** Primers used to perform real time PCR.

Gene target		Primer sequence	Annealing temperature
CCL20	Forward	5′-CTGGCTGCTTTGATGTCAGTG-3′	58°C
CCL20	Reverse	5′-GCAGTCAAAGTTGCTTGCTGC-3′	
hBD-2	Forward	5′-GCCTCTTCCAGGTGTTTTTG-3′	60°C
hBD-2	Reverse	5′-GAGACCACAGGTGCCAATTT-3′	
IL-23p19	Forward	5′-GGGACACATGGATCT AAGAG-3′	58°C
IL-23p19	Reverse	5′-GCAAGCAGAACTGACTGTTG-3′	
β-actin	Forward	5′-AAGATGACCCAGATCATGTTTGAGACC-3′	58 or 60°C
β-actin	Reverse	5′-AGCCAGTCCAGACGCAGGAT-3′	

### Isolation and Culture of Peripheral Blood Mononuclear Cells (PBMCs)

PBMCs were obtained from whole blood by density centrifugation on a Ficoll-Hypaque (GE Healthcare) gradient. RPMI-10 used for all cell cultures consisted of RPMI 1640 (Hyclone), 10% fetal calf serum (Hyclone), and *l*-glutamine, penicillin/streptomycin and sodium pyruvate (Gibco) added according to the manufacturers’ recommendations. CD4^+^ T cells were selected by positive selection (Miltenyi Biotec), and kept in RPMI-10 supplemented with 50 U/ml IL-2 (eBioscience) overnight before co-culture with antigen presenting cells.

**Figure 1:**
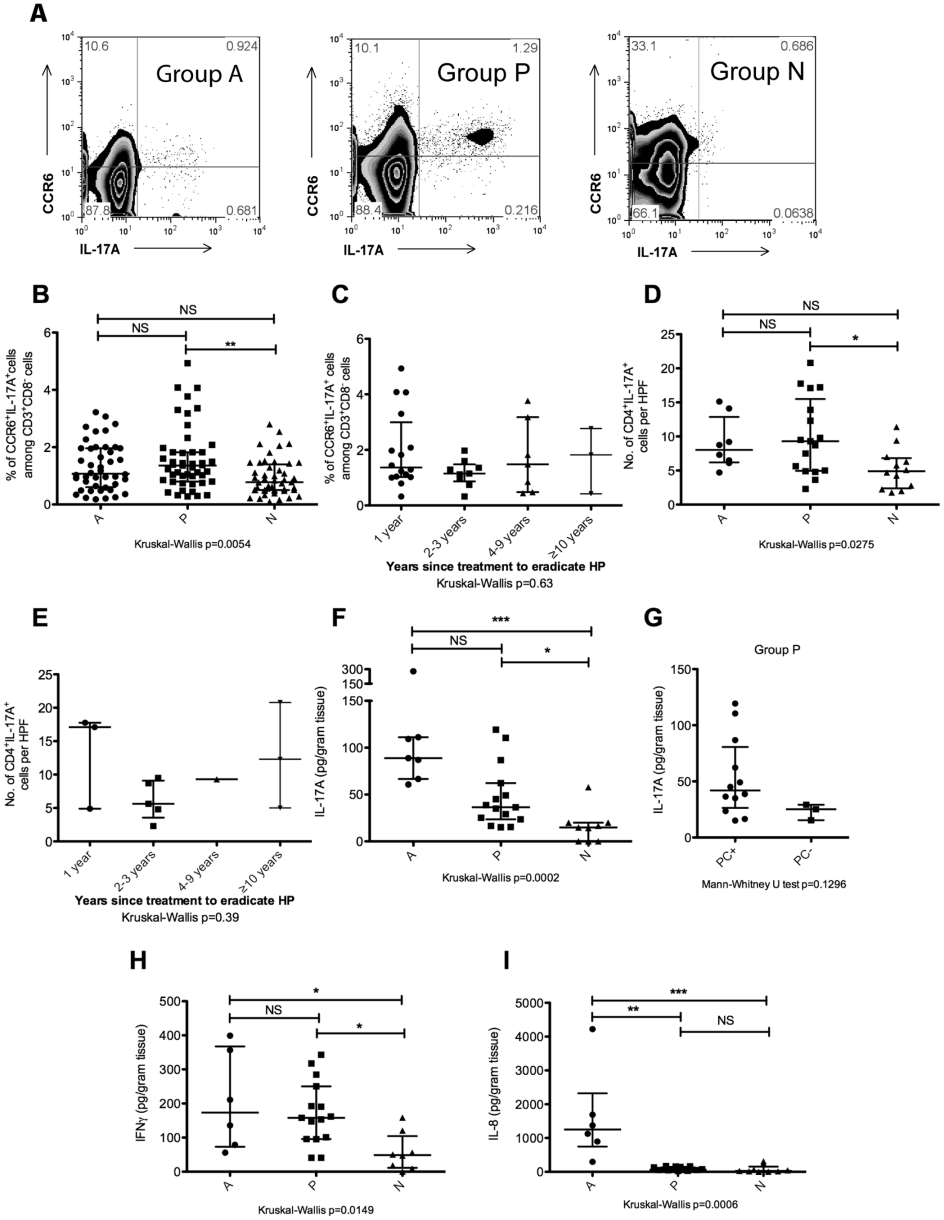
Elevated frequencies of cells that express IL-17A persist in individuals with past HP infection. (A) The percentage of CD3^+^CD8^−^CCR6^+^IL-17A^+^ cells as a function of CD3^+^CD8^−^ PBMCs was assessed by flow cytometry. PBMCs were activated with PMA and ionomycin for 5 hours in the presence of GolgiStop. Cells were stained for cell surface CD3, CD8, and CCR6, fixed, permeabilised, and stained for intracellular IL-17A. Th17 cells were defined as CCR6^+^IL-17A^+^ events within the CD3^+^CD8^−^ compartment. Representative flow cytometry plots of individuals from group A, P, and N have been depicted. (B) Scatter plot of CD3^+^CD8^−^CCR6^+^IL-17A^+^ cells as a percentage of CD3^+^CD8^−^ cells among PBMCs that had been stimulated with PMA and ionomycin. Group A (n = 44), group P (n = 47), and group N (n = 48). The median and interquartile ranges have been represented on the scatter plot as horizontal bars. (C) The frequency of CD3^+^CD8^−^CCR6^+^IL-17A^+^ events within the CD3^+^CD8^−^ compartment for individuals from group P divided according to years since HP treatment. 1 year (n = 13), 2–3 years (n = 9), 4–9 years (n = 7), and ≥10 years (n = 3). (D) Number of CD4^+^IL-17A^+^ cells per high powered field (HPF) in gastric biopsy samples. Immunofluorescence microscopy was performed on gastric biopsies obtained from 8 patients in group A, 17 patients in group P, 12 patients in group N. For each patient sample, ten HPFs were evaluated and the average number of CD4^+^IL-17A^+^ cells per HPF was represented on the scatter plot. (E) Number of CD4^+^IL-17A^+^ cells per HPF in samples from group P stratified according to years since HP treatment. 1 year (n = 3), 2–3 years (n = 5), 4–9 years (n = 1), and ≥10 years (n = 3). (F − I) Cytokine concentrations in clarified homogenate obtained from mechanically disrupted gastric biopsy samples were measured using the MILLIPLEX® xMAP® bead-based cytokine quantification assay. (F) IL-17A concentration in gastric biopsy samples obtained from patients in group A (n = 7), group P (n = 15), and group N (n = 9). (G) IL-17A concentration in gastric biopsy samples from group P individuals depicted in (F) who were further sub-grouped based on the presence (PC+) or absence (PC**−**) of histological evidence of pre-cancerous lesions (chronic atrophic gastritis or intestinal metaplasia) in the gastric mucosa. PC+ (n = 12), PC**−** (n = 3). (H) IFNγ concentration in gastric biopsy samples obtained from patients in group A (n = 7), group P (n = 15), and group N (n = 9). (I) IL-8 concentration in gastric biopsy samples obtained from patients in group A (n = 7), group P (n = 15), and group N (n = 9). NS: not significant, *p<0.05, **p<0.001, ***p<0.0001.

### Isolation and Expansion of Lamina Propria Mononuclear Cells (LPMCs)

Gastric biopsy samples were mechanically disrupted in a small volume of RPMI, centrifuged at 500 *g*, and clarified supernatant was collected for multiplex cytokine array analyses. The remaining tissue was digested and then placed in culture for 24 hours. Non-adherent cells containing LPMCs were collected, washed, and co-cultured with irradiated (40 Gy) allogeneic feeder cells (5 feeders : 1 LPMC) in 96-well plates containing RPMI-10 supplemented with 1 µg/ml phytohemagglutinin (PHA) (Sigma) and 200 U/ml IL-2. These cultures were stimulated with PHA and fresh allogeneic feeders every 7–8 days for 3 cycles, then harvested for assays.

**Figure 2:**
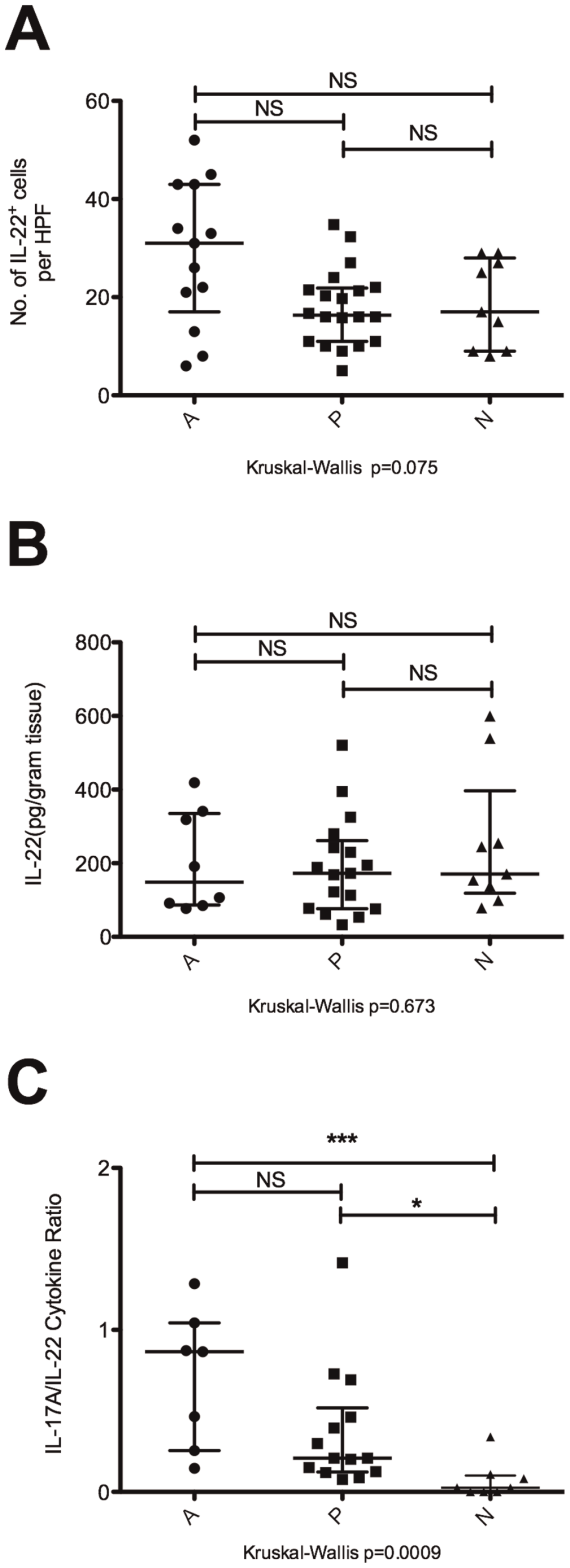
IL-22 expression in the gastric mucosa. (A) Number of IL-22^+^ cells per high powered field (HPF) in gastric biopsy samples. Immunofluorescence microscopy was performed on gastric biopsies obtained from 13 patients in group A, 20 patients in group P, and 9 patients in group N. Ten HPFs were evaluated per sample, and the average number of IL-22^+^ cells per HPF was represented on the scatter plot. (B) *Ex vivo* concentration of IL-22 in gastric biopsies. Cytokine concentrations in clarified homogenate obtained from mechanically disrupted gastric biopsy samples were measured by ELISA. Group A (n = 8), group P (n = 17), and group N (n = 9). (C) The ratio of gastric mucosal IL-17A to IL-22 was determined by dividing the concentration of IL-17A in gastric mucosal homogenate with the concentration of IL-22 found in the same biopsy sample obtained from a given individual. Group A (n = 7), group P (n = 14), and group N (n = 8). NS: not significant, *p<0.05, ***p<0.0001.

### HP Lysate and Antigen-pulsing

Lysate from HP strain 26695 was prepared by sonication and used at 10 µg/ml for all assays. PBMCs used as antigen-presenting cells (APCs) in co-culture assays were pulsed for 24 hours with lysate and irradiated at 40 Gy prior to co-culture. Purified no azide low-endotoxin mouse anti-human HLA-DR, DP, DQ antibody (Becton Dickinson, clone Tü39) was used to block antigen presentation by MHC Class II. In some experiments, recombinant human IL-1 receptor antagonist (R&D Systems) was used at a final concentration of 200 ng/ml to block the activity of IL-1β in culture.

### Intracellular Staining and Flow Cytometry

PBMCs were stimulated for 5 hours in RPMI-10 with 1 µM phorbol 12-myristate 13-acetate (PMA) (Sigma), 1 µg/ml ionomycin (Sigma), and Golgistop (BD Pharmingen), then fixed and permeabilised (Cytofix/Cytoperm kit, Becton Dickinson), and stained with anti-IL-17A-FITC, anti-CCR6-PE, anti-CD3-PE-Cy7, anti-CD8-allophycocyanin-eFluor780 (eBioscience). Flow cytometry was performed on a CyAN analyzer (Beckmann Coulter). CD4^+^ T cells were gated as CD3^+^CD8^−^ events because cell surface expression of CD4 is downregulated during stimulation of PBMCs with PMA and ionomycin, which makes it difficult to unambiguously identify CD4^+^ T cells using CD4 as a cell marker.

### Immunofluorescent Staining of Frozen Sections

Antral biopsies were snap frozen in Tissue-Tek O.C.T. compound (Sakura Finetek) and stored at −80°C. 5 µm sections were cut, fixed in ice-cold acetone, and blocked using either PBS 0.5% BSA (Sigma) or a biotin/streptavidin blocking kit (Vector Labs) according to the manufacturer’s instructions. The following antibodies were used to stain sections: mouse anti-IL-17A-biotin (eBioscience), streptavidin-Alexa Fluor 546 (Invitrogen), polyclonal goat anti-IL-22 (R&D), donkey anti-goat IgG Alexa Fluor 546 (Invitrogen), and mouse anti-CD4 Alexa Fluor 488 (eBioscience). Antibodies were diluted in PBS containing 0.5% human AB serum (Sigma) for incubating with sections, and washing was done in PBS. Sections were counterstained with DAPI before mounting in fluorescent mounting medium. Samples were imaged with an AxioCam fluorescent microscope (Zeiss), and images were analysed using Axiovision LE software (Zeiss).

**Figure 3:**
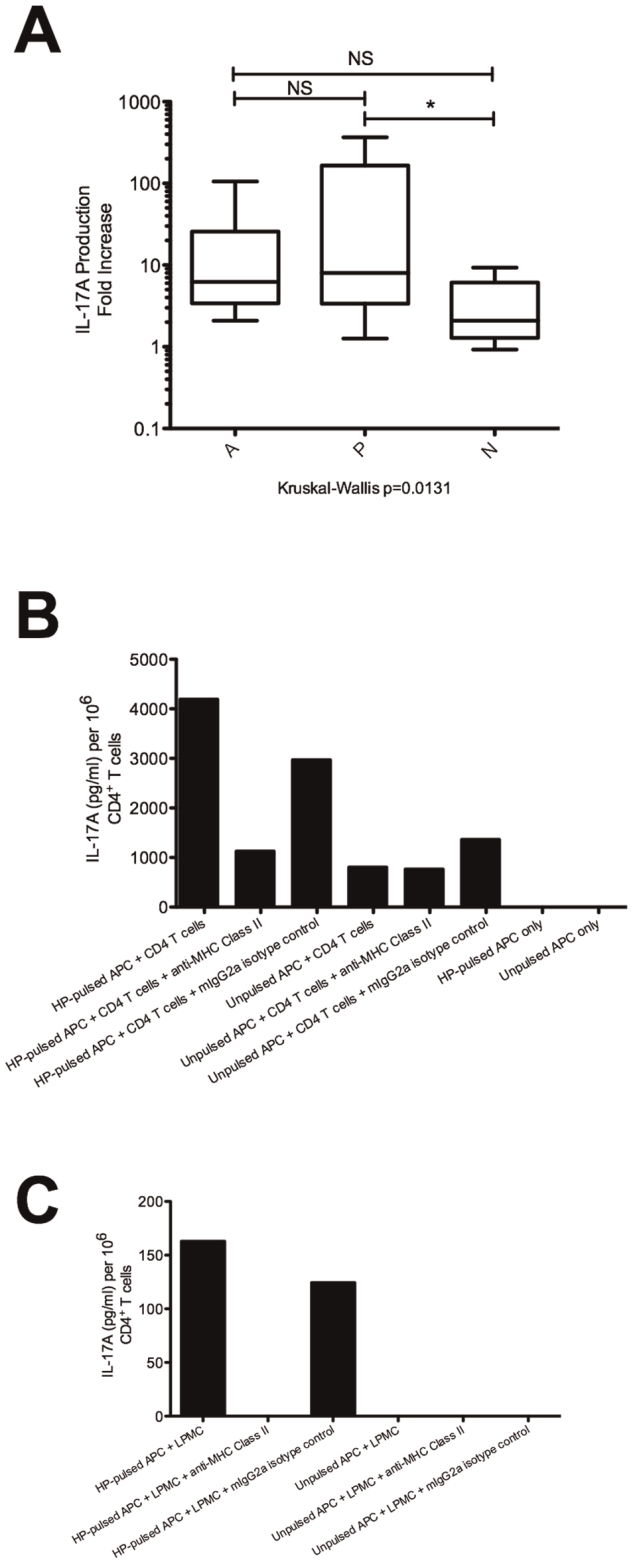
Persistent HP-specific Th17 responses in individuals with past HP infection. (A) The IL-17A response of PBMCs to HP lysate. 2×10^6^ PBMCs from the three groups of patients in this study were incubated for 72 hours in a 96-well plate, together with 10 µg/ml whole bacterial lysate prepared from HP strain 26695. Subsequently, culture supernatant was harvested and assayed for IL-17A production by ELISA. The fold increase in IL-17A production is depicted, i.e. (cytokine concentration in wells containing PBMCs stimulated with HP lysate) ÷ (cytokine concentration in control wells containing unstimulated PBMCs). Group A (n = 7), group P (n = 17), and group N (n = 10). NS: not significant, *p<0.05. (B) The HP-specific IL-17A response of CD4^+^ T cells purified from PBMCs. PBMCs were pulsed with HP strain 26695 lysate for 24 hours, and then irradiated at 40 Gy. These cells were used as APCs, and co-cultured for 48 hours with purified autologous CD4^+^ T cells at a ratio of 5 APC: 1 CD4^+^ T cell. In wells where MHC class II blocking antibody (10 µg/ml) was used, antibody was incubated with APCs for 1 hour prior to addition of responder CD4^+^ T cells. In control wells, APCs were incubated with an equal concentration of mouse IgG2a isotype control antibody for the same length of time. IL-17A levels in culture supernatant were measured using ELISA. The response from one representative group P patient has been depicted. (C) The HP-specific IL-17A response of gastric mucosal LPMCs. HP-pulsed PBMCs were prepared as in (B) for use as APCs. Autologous LPMCs expanded from gastric biopsy samples were used as responders, and IL-17A levels in culture supernatant were measured by ELISA after 48 hours of co-culture. The response from one representative group P patient has been depicted.

**Table 3:** The IL-17A response of CD4^+^ PBMCs to HP-pulsed APCs.

	Active	Past	Naive
IL-17A production per 10^6^ CD4^+^ T cells (pg/ml)	2594±686, n = 3	2576±629, n = 8	431±137, n = 3
Mean fold-decrease in the presence of MHCclass II blocking antibody	2±0.3	5±3	1±0.3

The mean ± SEM (standard error of mean) has been depicted for measurements of IL-17A concentration in the supernatant of co-culture experiments performed as described in the legend for figure 3B. CD4^+^ T cells were co-cultured with autologous HP-pulsed APCs either in the absence or presence of MHC Class II blocking antibody. The fold-decrease in response was calculated by dividing the supernatant concentration of IL-17A from the culture without MHC Class II blockade, by the IL-17A concentration in the culture with MHC Class II blockade. The mean fold-decrease was obtained for the indicated number of biological replicates in each group, and mean ± SEM has been reported in the table.

**Table 4:** The IL-17A response of expanded LPMCs to HP-pulsed APCs.

	Active	Past	Naive
Mean IL-17A production per 10^6^ LPMCs(pg/ml)	860±546, n = 3	169±38, n = 4	18±8, n = 4
Mean fold-decrease in the presence of MHCclass II blocking antibody	805±581	73±41	14±7

The mean ± SEM (standard error of mean) has been depicted for measurements of IL-17A concentration in the supernatant of co-culture experiments performed as described in the legend for figure 3C. LPMCs were co-cultured with autologous HP-pulsed APCs, either in the absence or presence of MHC Class II blocking antibody. The fold-decrease in response was calculated by dividing the supernatant concentration of IL-17A from the culture without MHC Class II blockade, by the IL-17A concentration in the culture with MHC Class II blockade. The mean fold-decrease was obtained for the indicated number of biological replicates in each group, and mean ± SEM has been reported in the table.

### mRNA Isolation and Real Time PCR

mRNA was isolated from gastric biopsy samples (RNA-Protein isolation kit, Macherey-Nagel), converted to cDNA (Superscript III, Invitrogen), and gene expression was evaluated by real time PCR using SYBR green chemistry. Primers used and their annealing temperatures have been summarized in table 2. For all reactions, elongation was carried out at 72°C, and 40 cycles of amplification were performed.

### Cytokine Quantification

HP-specific IL-17A or IFNγ levels in culture supernatants, and IL-22 concentrations in biopsy supernatant samples, were assayed using ELISA kits (eBioscience). Milliplex xMAP kits (Millipore) were used to measure all other cytokines.

### Statistical Analyses

The Kruskal-Wallis test with Dunn’s multiple comparisons post-hoc test was used for statistical analyses of data from the 3 patient groups (Groups A, P, and N). The Mann Whitney U test was used when making comparisons between two groups of samples. Data analysis was performed using GraphPad Prism software (GraphPad Software Inc.).

**Figure 4:**
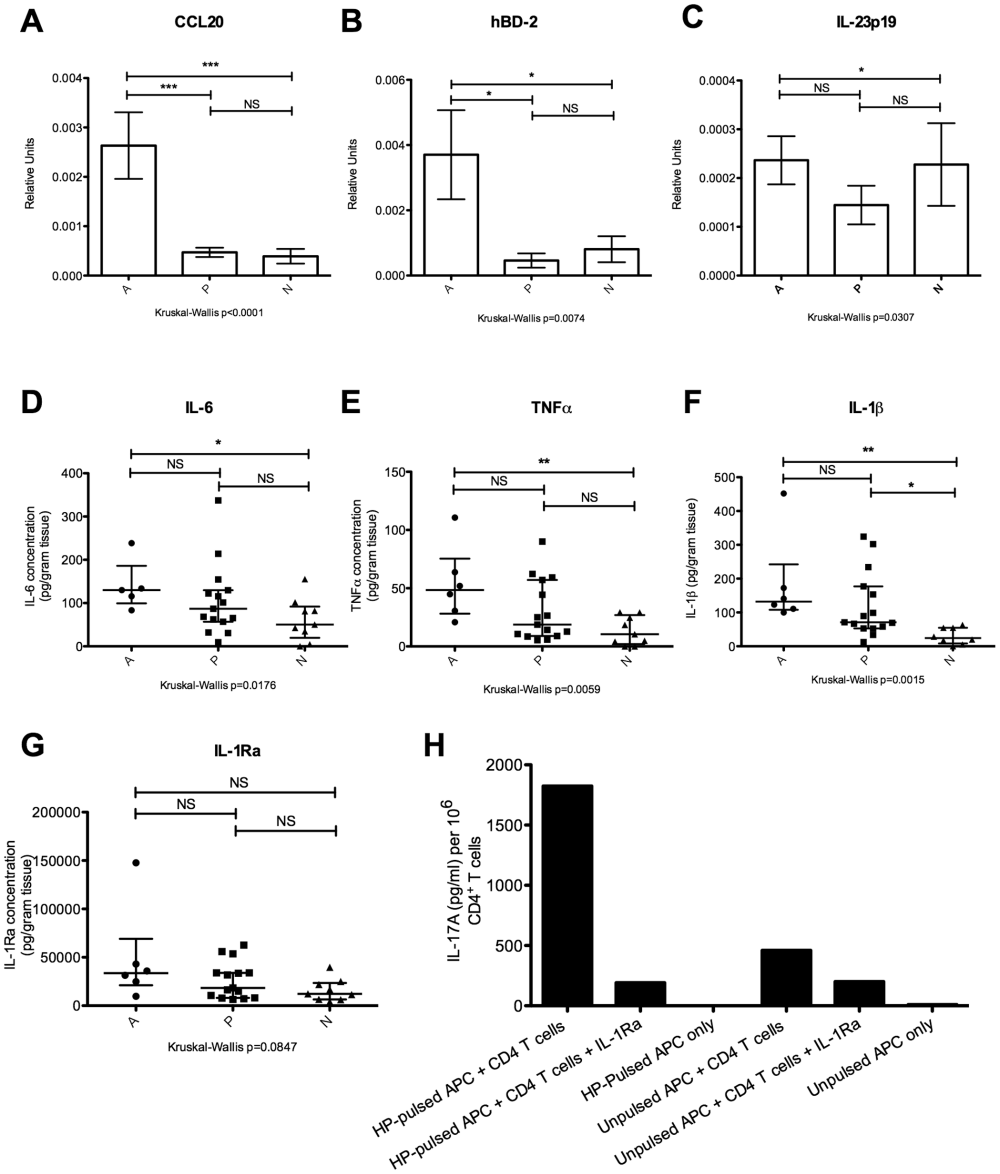
Gastric mucosal expression of cytokines that modulate Th17 responses. (A) Semi-quantitative real-time PCR was used to determine gene expression of CCL20 in gastric biopsy samples, normalised to expression of β-actin. Group A (n = 17), group P (n = 27), group N (n = 20). (B) Semi-quantitative real-time PCR was used to determine gene expression of hBD-2 in gastric biopsy samples, normalised to expression of β-actin. Group A (n = 16), group P (n = 11), and group N (n = 12). (C) Semi-quantitative real-time PCR was used to determine gene expression of IL-23p19 in gastric biopsy samples, normalised to expression of β-actin. Group A (n = 15), group P (n = 31), and group N (n = 30). (D – G) *Ex vivo* protein concentrations of IL-6, TNF-α, IL-1β, and IL-1Ra in homogenised gastric biopsy samples were measured by MILLIPLEX® xMAP® bead-based cytokine quantification assay. Group A (n = 6), group P (n = 14), and group N (n = 7). (H) IL-1 receptor antagonist blocks IL-17A production by CD4^+^ T cells co-cultured with HP-pulsed APCs. PBMCs obtained from a group P individual were pulsed with HP and prepared as in figure 3B for use as APCs. These APCs were co-cultured for 48 hours with purified autologous CD4^+^ T cells at a ratio of 5 APCs: 1 CD4^+^ T cell, either in the absence or presence of IL-1Ra (final concentration 200 ng/ml). IL-17A levels in culture supernatant were measured using ELISA. Representative data from 1 of 2 independent experiments has been depicted. NS: not significant, *p<0.05, **p<0.001, ***p<0.0001.

## Results

### IL-17A Responses Remained Elevated in the Blood and Gastric Mucosa of Individuals with Evidence of Previous *H. pylori* Infection

The frequencies of IL-17A-secreting CD4^+^ T cells in PBMCs that had been stimulated with PMA and ionomycin were measured by flow cytometry, gating on CD3^+^CD8^−^ cells that were also CCR6^+^ and IL-17A^+^ (figure 1A). Individuals with evidence of past HP infection but who no longer had ongoing infection (group P) exhibited elevated frequencies of CD3^+^CD8^−^CCR6^+^IL-17A^+^ cells in the blood (median 1.4%, range 0.3–4.9%) that was statistically different from individuals who were naive to HP (group N, median 0.8%, range 0.1–2.8%) (figure 1B). Levels of peripheral blood CD3^+^CD8^−^CCR6^+^IL-17A^+^ cells in individuals with active, ongoing HP infection (group A, median 1.1%, range 0.2–3.2%) were not statistically different from the other two groups. The frequency of CD3^+^CD8^−^CCR6^+^IL-17A^+^ cells among CD3^+^CD8^−^ PBMCs remained elevated in some individuals from group P even if they had been treated for HP more than 10 years ago (figure 1C). Frozen sections prepared from biopsy samples were also examined by immunofluorescence microscopy to identify CD4^+^IL-17A^+^ cells in the gastric mucosa. Representative immunofluorescence microphotographs showing samples from group A, group P, and group N can be found in figures S1A–S1C respectively. Gastric mucosal CD4^+^IL-17A^+^ cells were significantly elevated in group P patients compared to group N (figure 1D). The median number of infiltrating CD4^+^IL-17A^+^ cells was 8.0 (range 4.7–15.1) in group A, 9.3 (range 2.3–20.8) in group P, and 4.9 (range 1.8–11.4) in group N. Gastric mucosal CD4^+^IL-17A^+^ cells remained persistently elevated in some individuals from group P even if they had been treated for HP more than 10 years ago (figure 1E). Representative microphotographs depicting gastric mucosal samples obtained from a patient 2–3 years after treatment to eradicate HP, and from a patient who had received treatment to eradicate HP>10 years ago can be found in figures S1D and S1E respectively. IL-17A protein levels were also significantly higher in gastric biopsy samples from group A (89 pg/ml, range 61–278) and group P (36 pg/ml, range 15–119), compared to group N (15 pg/ml, range 0–58) (figure 1F). Group P individuals depicted in figure 1F were further divided into 2 sub-groups based on the presence or absence of pre-cancerous changes (PC) on histology: PC+ individuals had evidence of chronic atrophic gastritis or intestinal metaplasia in the gastric mucosa, whereas PC**−** individuals did not. PC+ individuals in group P tended to have higher levels of gastric mucosal IL-17A than PC**−** individuals, although the difference did not reach statistical significance possibly due to the small size of the PC**−** sub-group (figure 1G).

IFNγ is produced by Th1 CD4^+^ T cells. The persistence of IFNγ-producing cells in our cohort of patients was also examined. Frequencies of IFNγ+ cells among CD3^+^CD8^−^ PBMCs following stimulation with PMA and ionomycin were not significantly different across groups A, P, and N (figure S2). However, gastric mucosal IFNγ levels were significantly higher in groups A (173 pg/ml, range 56–399) and P (158 pg/ml, range 41–343) compared to group N (49 pg/ml, range 0–159) (figure 1H).

Altogether, the observations depicted in figures 1C−1H suggest that gastric mucosal inflammation persists in group P patients despite the absence of ongoing HP infection, and identify CD4^+^IL-17A^+^ cells as a component of this persistent inflammation.

Ongoing HP infection in our cohort of individuals with past HP infection was excluded on the basis of negative findings in gastric biopsies sent for histological examination (6 sites), culture (2 sites) and testing for urease activity (1 site). Despite sampling multiple independent gastric mucosal sites to arrive at this conclusion, the apparent persistence of gastric mucosal CD4^+^IL-17A^+^ cells in patients from group P might still be due to ongoing infection with HP at localised sites that were missed during endoscopy. IL-8 is released during acute gastric mucosal inflammation associated with ongoing HP infection. [9] Since this is a diffusible cytokine, we surmised that ongoing HP infection might be revealed by elevated gastric mucosal levels of IL-8 even if the actual site of infection was missed during endoscopic sampling. Increased levels of gastric mucosal IL-8 were observed in group A patients (1250 pg/ml, range 297–4223) compared to group N (27 pg/ml, range 0–312) (figure 1I). However, IL-8 levels in samples from group P (65 pg/ml, range 24–171) were significantly lower than samples from group A and not significantly different than samples from group N. This suggests that gastric inflammation caused by undiagnosed ongoing HP infection is an unlikely explanation for the persistence of CD4^+^IL-17A^+^ cells that was observed in group P.

### Gastric Mucosal IL-22 Levels did not Differ Significantly Across the Three Groups of Patients

A subset of IL-17A expressing cells also secrete IL-22, a cytokine that enhances epithelial integrity and regeneration in the gut, but promotes inflammation under certain experimental settings. [24] Gastric mucosal IL-22 expression in our patient cohort was assayed using immunofluorescence microscopy and ELISA. IL-22 expression did not differ significantly among our 3 groups of patients (figures 2A and 2B). IL-22 has been reported to have tissue protective as well as pro-inflammatory properties; the balance between these two activities might be governed by the extent of IL-22 co-expression with IL-17A. IL-22 in the absence of IL-17A has tissue-protective effects; however IL-22 and IL-17 synergistically provoke inflammation when co-expressed. [25] As a measure of cytokine co-expression, the ratio of IL-17A concentration to IL-22 concentration in the supernatant of mechanically disrupted gastric biopsy samples was calculated for individual biopsy samples. IL-17A/IL-22 ratios were significantly elevated in samples from groups A and P compared to group N (figure 2C). Co-expression of IL-17A and IL-22 suggests that the gastric cytokine milieu in group P patients continues to be biased against the baseline state of mucosal maintenance found in HP-naïve patients, despite the absence of ongoing HP infection.

### HP-specific Th17 Responses Persist in Patients with Evidence of Past HP Infection

In order to study HP-specific IL-17A responses, whole lysate of HP strain 26695 was used to stimulate PBMCs and the responses were normalised against PBMCs that had not been pulsed with HP lysate. PBMCs from individuals in group P produced a median 8-fold more IL-17A when stimulated with HP than control PBMCs from the same individuals that had not been pulsed with HP lysate (range 1–367), which was significantly higher than the median 2-fold increase in group N (range 0.9–9) (figure 3A). PBMCs from group A produced a median 6-fold more IL-17A when stimulated with HP (range 2–105), but the difference compared to group N did not reach statistical significance. In addition to CD4^+^ T cells, several other subsets of immune cells found in PBMCs have been reported to secrete IL-17A, e.g. CD8^+^ T cells, NK cells, NKT cells, γδ TCR T cells. [26] Instead of unfractionated PBMCs, the experiment was repeated using purified CD4^+^ T cells as responders to demonstrate that these cells mediated the persistent HP-specific responses in group P. The response of a representative patient from group P has been depicted in figure 3B. Purified CD4^+^ T cells co-cultured with HP-pulsed APCs produced a detectable IL-17A response. This HP-specific IL-17A response decreased ∼3 fold in the presence of antibody that blocked antigen presentation by MHC Class II compared to isotype control mouse IgG2a antibody. The experiment was repeated for a number of individuals from the 3 patient groups, and the results have been summarized in table 3. IL-17A production was higher in PBMCs from groups A and P than from group N, and blocking MHC Class II resulted in decreased IL-17A production.

To demonstrate the persistence of HP-specific Th17 cells in gastric mucosal samples from patients with past HP infection, the experiment was performed using gastric LPMCs as responders. Since only limited numbers of LPMCs could be recovered from biopsy samples, it was necessary to expand gastric LPMCs for 3 weeks before sufficient cells for our assays could be obtained. The IL-17A response of a representative patient from group P has been depicted in figure 3C. LPMCs stimulated with autologous HP-pulsed APCs released IL-17A into culture supernatant. In the presence of antibody that blocked antigen presentation by MHC Class II, this LPMC response decreased to undetectable levels (limit of detection: 4 pg/ml). The experiment was repeated for several individuals from groups A, P, and N, and the results have been summarized in table 4. Levels of IL-17A well above the limit of detection for the ELISA were observed when LPMCs from group A or group P were co-cultured with autologous HP-pulsed APCs, and MHC Class II blockade reduced the production of IL-17A.

In summary, we observed the persistence of HP-specific, IL-17A-producing CD4^+^ T cells that were restricted by MHC Class II among PBMCs as well as LPMCs derived from group P samples. Since the other known cellular sources of IL-17A are not restricted by MHC Class II, [26] these results suggest that the persistent IL-17A responses in individuals from group P are mainly mediated by Th17 cells.

### Gastric Mucosal IL-1β Levels Remain Elevated in Patients with Past HP Infection

To identify factors responsible for the persistence of Th17 cells in the gastric mucosa of group P patients, expression levels of cytokines related to the generation, maintenance, or recruitment of Th17 cells were assessed. CCL20 is a ligand for the chemokine receptor CCR6, which is expressed on Th17 cells. [27], [28] Consistent with previous reports, [29], [30] CCL20 gene expression was significantly elevated in the gastric mucosa of patients with ongoing HP infection (figure 4A). However, CCL20 gene expression was not significantly increased in group P, suggesting that recruitment of CCR6^+^ Th17 cells into the gastric epithelium by CCL20 was not responsible for the persistence of gastric infiltrating Th17 cells in this group. Human β-defensin-2 (hBD-2) can serve as an alternative ligand for CCR6. [31], [32] However, gastric mucosal mRNA levels of hBD-2 were also not significantly elevated in group P, but only in group A (figure 4B). IL-23 is important for the maintenance of human Th17 cells. [12] Although gene expression of IL-23p19 was significantly higher in group A compared to group N, it was not significantly increased in gastric mucosal samples from group P (figure 4C). The pro-inflammatory cytokines IL-6 and TNF-α are important for the differentiation of Th0 cells into Th17 cells. [12] Significantly higher concentrations of these cytokines were only observed in gastric mucosal samples from group A compared to group N, but not in group P (figures 4D and 4E). IL-1β influences the biology of Th17 cells in a variety of ways, including the differentiation, [33] and proliferation of these cells. [34] IL-1β also stimulates the release of IL-17A by Th17 cells. [35] Interestingly, significantly higher concentrations of IL-1β were observed in gastric biopsy samples from both group A and group P compared to group N (figure 4F). In the same samples, concentrations of IL-1 receptor antagonist (IL-1Ra) were ∼100 fold higher than IL-1β, but did not differ significantly across the 3 groups (figure 4G). To determine whether there was a functional association between IL-1β and the Th17 response, the experiment depicted in figure 3B was repeated using recombinant IL-1Ra to neutralise the biological activity of IL-1β. Purified CD4^+^ T cells from a group P individual produced less IL-17A when co-cultured with autologous HP-pulsed APCs in the presence of IL-1Ra (figure 4H). This suggests that the action of IL-1β is important for secretion of IL-17A by *H. pylori*-specific CD4^+^ T cells, and that IL-1β plays a functional role in the persistent Th17 response observed in group P patients.

## Discussion

Previous studies have demonstrated the persistence of gastric inflammation following HP eradication in an animal model, [36] as well as in humans. [22] Other studies have shown that IL-17A responses are present during ongoing HP infection, although the nature of these IL-17A producing cells was not characterized. Based on the findings presented in this study, we conclude that IL-17A responses persist in individuals with past HP infection, and are mediated largely by Th17 cells.

In order to perform this study, individuals with past but not ongoing HP infection were identified. Individuals so classified had either a history of treatment to eradicate HP, or tested seropositive for HP. These individuals were also carefully screened to exclude ongoing HP infection. The gastric epithelium of each individual was sampled at nine independent sites (antrum, incisura, body, and cardia), and biopsies were sent to exclude the presence of HP by several methods (6 samples were sent for histology, 2 for culture, and 1 for urease test). Gastric mucosal levels of IL-8 were also assayed, since this is a diffusible cytokine that is released during ongoing HP infection. The low levels of IL-8 observed in group P, comparable to levels in group N (figure 1I), suggest that individuals from group P were not likely to harbour ongoing HP infection at localised sites in the stomach that were inadvertently missed during endoscopic sampling. Based on these criteria, we concluded that the individuals in group P did not have ongoing HP infection at the time of sampling for our study.

Individuals from group P showed persistent IL-17A responses mediated by MHC Class II-restricted CD4^+^ T cells that were specific for HP, despite absence of ongoing HP infection. Previous studies have shown that lymphocytes persist in the gastric epithelium after resolution of HP infection in animal models of infection, [36] as well as in humans. [22] We have extended previous findings by demonstrating that IL-17A responses contribute to chronic gastric inflammation in individuals with past HP infection, and this is primarily due to the persistence of HP-specific Th17 cells (figures 1D and 3C). Since several different immune cell types can secrete IL-17A, [26] it was necessary to identify Th17 cells as the main cells responsible for the persistent IL-17A responses. Th17 cells in frozen sections of gastric epithelium were identified as cells that stained for both CD4 and IL-17A (figure 1D). Purified autologous CD4^+^ T cells were used as effectors in the assay depicted in figure 3B, and blocking antibodies were used to demonstrate that the responses were restricted by MHC Class II. MHC Class II restriction of IL-17A responses was also demonstrated for the LPMC experiment depicted in figure 3C. Based on these results, we conclude that Th17 cells are the main producers of IL-17A in the blood and lamina propria of individuals with active HP infection as well as those with past HP infection, although we cannot exclude a minor contribution from cells that are not restricted by MHC Class II, e.g. CD8^+^ T cells, γδ TCR T cells, NK cells, or NKT cells. These cells might be responsible for the small residual IL-17A responses that remained after MHC Class II blockade (figure 3B).

It is unclear whether these persistent Th17 responses simply represent the activity of effector memory T cells that persist by homeostatic proliferation following HP clearance. This might be addressed by an in depth assessment of cellular phenotype (e.g. CD45RA, CD45RO, CD62L, CD27, CD28, CCR6, etc). However, in an animal model where timed eradications were performed following HP infection, eradication of HP after 4 weeks of infection resulted in the absence of persistent gastric lymphocytic infiltrate whereas persistent lymphocytic infiltrate was observed when HP was eradicated after 8 weeks of infection. [36] Since effector memory T cells should have developed in both scenarios, the development of detectable persistent inflammation only after more prolonged infection suggests that factors in addition to homeostatic proliferation may be triggered to maintain gastric LPMCs during chronic HP infection. Once activated, we speculate that these mechanisms might persist following HP eradication and continue to support gastric inflammation in the absence of HP antigen. We explored possible mechanisms that might maintain gastric mucosal Th17 responses. Continual migration of lymphocytes from blood into the gastric epithelium might maintain Th17 numbers in the gastric epithelium. However, gastric epithelial expression of CCL20 and hBD-2, both chemotactic ligands for CCR6 that is expressed on Th17 cells, was not elevated in biopsy samples from group P (figures 4A and 4B). Th17 cells might be maintained by IL-23, or by their continual differentiation from Th0 cells driven by exposure to IL-1β, IL-6, TNF-α, and TGF-β. [12] Levels of IL-23p19, IL-6, and TNF-α were not significantly elevated in individuals with past HP infection (figures 4C–4E). Expression of TGF-β was high and not significantly different across all 3 groups of individuals (data not shown). However, IL-1β protein levels remained significantly elevated in the gastric mucosa of individuals from group P (figures 4F). We speculate that the presence of IL-1β in the gastric environment might favor low-level proliferation of Th17 cells previously recruited into the gastric mucosa during active HP infection, [34] and so maintain the presence of Th17 cells even after resolution of HP infection. Alternatively, IL-1β has been shown to trigger the release of IL-17A by Th17 cells. [35] Consistent with these hypotheses, neutralization of IL-1β with recombinant human IL-1Ra reduced the HP-specific IL-17A response of purified CD4^+^ T cells to autologous HP-pulsed APCs in a 48 hour co-culture assay (figure 4H). Polymorphisms in *IL1B* or *IL1RN* genes that increase circulating levels of IL-1β have been linked to the development of HP-associated disease, [37], [38] although the mechanism for this association remains unclear. We speculate that genetic propensity to over-produce IL-1β might increase chronic inflammation in the gastric mucosa caused by Th17 cells. Nevertheless, the factors that persistently upregulate IL-1β expression in the gastric mucosa despite lack of ongoing HP infection remain unresolved. IL-17A itself can stimulate the release of IL-1β, although this occurs in the setting of acute inflammation associated with secretion of other pro-inflammatory cytokines, e.g. IL-6, IL-8, TNF-α, [12] and has not been reported for chronic inflammation. Gastric mucosal levels of IL-1Ra, which acts as an antagonist for IL-1β, were not significantly different across our three groups of patients (figure 4G).

Experimental evidence from animal models, [36] as well as epidemiological evidence from follow-up studies in humans, [39], [40] suggest that the risk of developing gastric adenocarcinoma is not completely eliminated after HP eradication. We speculate that our findings might relate to this persistent risk of cancer. Accumulating evidence implicates IL-17A and Th17 responses in the pathogenesis of gastric cancer. The IL-17A G197A allele has been associated with elevated IL-17A secretion and increased risk of cancer. [41] A different study reported that the A7488G polymorphism in IL-17F, another cytokine in the IL-17 family, was more highly associated with elevated risk of gastric cancer. [42] Higher frequencies of Th17 cells have been observed in the blood of gastric cancer patients compared with healthy controls, and the proportion of Th17 cells increased with cancer stage in the blood as well as within tumor-draining lymph nodes. [43] The frequency of Th17 cells was significantly elevated in tumor-infiltrating lymphocytes derived from early gastric cancer samples compared with normal gastric mucosa. [20] Furthermore, increased angiogenesis has been associated with high gene expression of IL-17A in gastric tumors. [19] When placed in the context of these reports, our study fills a gap in the literature concerning the timeline of Th17 responses during HP infection. Elevated Th17 responses may not be a late manifestation of gastric cancer, but are perhaps a direct continuation of persistent HP-specific Th17 responses that may contribute to the pathogenesis of gastric cancer from early in the timeline of HP infection. Our results suggest that even after eradication of HP, these Th17 responses might persist. Individuals from group P with pre-cancerous histological lesions in the gastric mucosa had higher persistent levels of IL-17A than group P individuals without pre-cancerous lesions, although this difference was not statistically significant (figure 1G). We hypothesise that chronic IL-17A signaling may promote carcinogenesis, perhaps in synergy with co-expressed IL-22 (figure 2C), by driving persistent expression of anti-microbial peptides and matrix metalloproteinases in the gastric mucosa. [12], [24] Elevated expression of these IL-17A target genes facilitates epithelial-mesenchymal transition, [44], [45] and has been associated with poor prognosis in gastric cancer. [46], [47] Ongoing experiments are underway to determine whether IL-17A and IL-22 co-signaling regulates gene expression in ways relevant to the pathology of HP-associated disease. We speculate that in addition to the eradication of HP infection, pharmacological suppression of gastric mucosal Th17 responses, perhaps by IL-1β blockade, might also prove helpful for preventing the long-term complications of HP infection.

## Supporting Information

Figure S1 **Representative immunofluorescence microphotographs.** Immunofluorescence microscopy was performed on gastric mucosal samples that had been stained for CD4, IL-17A, and DAPI. Representative microphotographs of samples from (A) group A, (B) group P, and (C) group N. Representative microphotographs of samples obtained from (D) a patient 2–3 years after treatment to eradicate H. pylori, and (E) from a patient who had received treatment to eradicate H. pylori infection >10 years ago. White arrows on the merged microphotographs indicate CD4+IL-17A+ cells.
(TIF)

Figure S2 **Percentage of IFNγ+ cells among CD3+CD8− cells following stimulation of PBMCs with PMA and ionomycin.** PBMCs were activated with PMA and ionomycin for 5 hours in the presence of Golgistop, stained for cell surface CD3 and CD8, fixed, permeabilised, stained for intracellular IFNγ, and analysed using flow cytometry. (A – C) Cells have been gated on CD3+CD8− events. The FACS plots depict the IFN? response among CD3+CD8− T cells of representative patients from groups A, P, and N respectively. (D) Summary of data points from all patient samples analysed: group A (n = 37), group P (n = 44), and group N (n = 40). NS: not significant.
(TIF)
