# Supplementary figures and images for: Persistent Helicobacter pylori Specific Th17 Responses in Patients with Past H. pylori Infection Are Associated with Elevated Gastric Mucosal IL-1β
Source: PLoS One. 2012 Jun 25;7(6):e39199. doi: 10.1371/journal.pone.0039199 (PMC3382622; doi:10.1371/journal.pone.0039199)

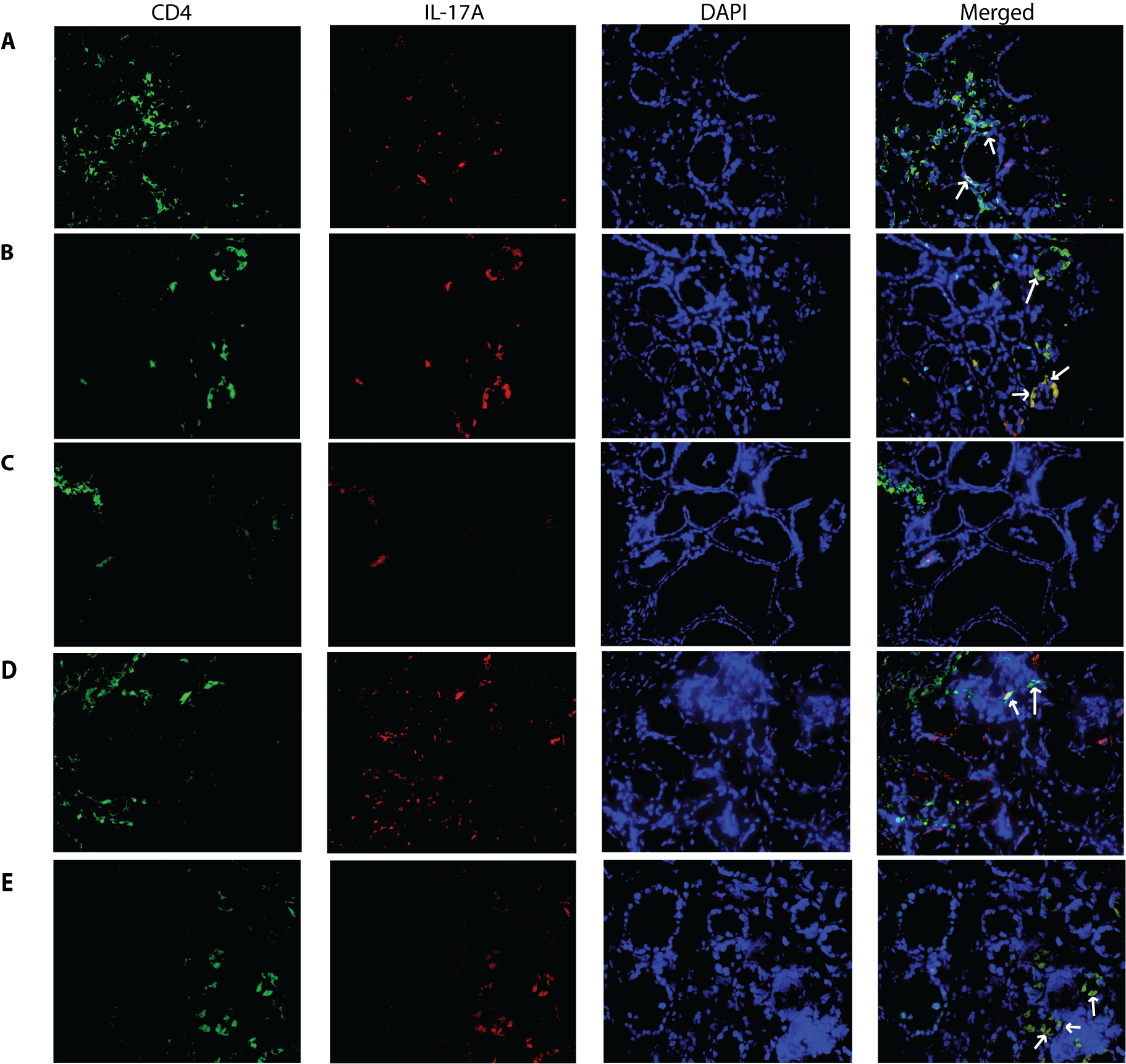

Supplement: Figure S1 — Representative immunofluorescence microphotographs. Immunofluorescence microscopy was performed on gastric mucosal samples that had been stained for CD4, IL-17A, and DAPI. Representative microphotographs of samples from (A) group A, (B) group P, and (C) group N. Representative microphotographs of samples obtained from (D) a patient 2–3 years after treatment to eradicate H. pylori, and (E) from a patient who had received treatment to eradicate H. pylori infection >10 years ago. White arrows on the merged microphotographs indicate CD4+IL-17A+ cells. (TIF) [file pone.0039199.s001.tif]

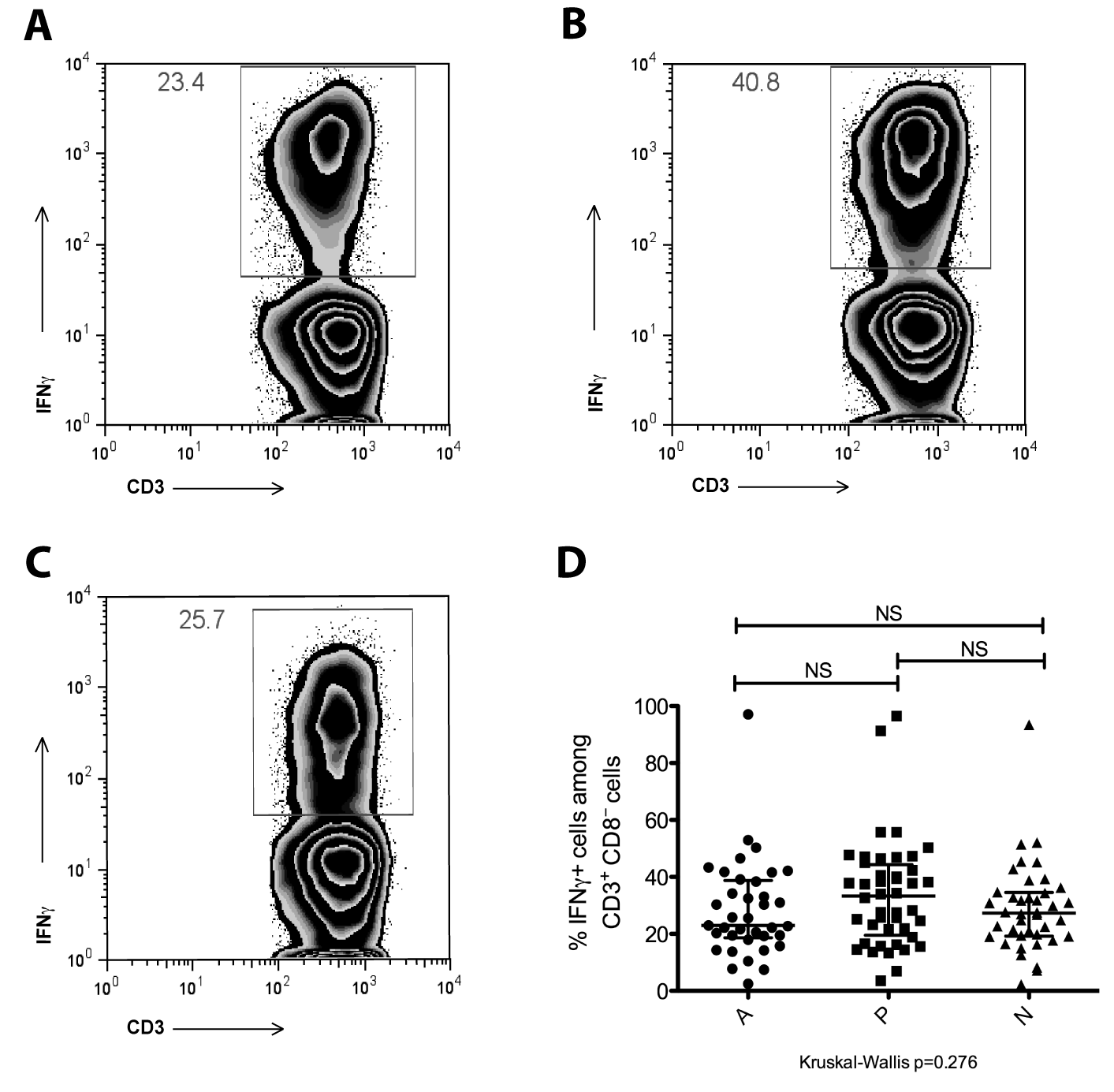

Supplement: Figure S2 — Percentage of IFNγ+ cells among CD3+CD8− cells following stimulation of PBMCs with PMA and ionomycin. PBMCs were activated with PMA and ionomycin for 5 hours in the presence of Golgistop, stained for cell surface CD3 and CD8, fixed, permeabilised, stained for intracellular IFNγ, and analysed using flow cytometry. (A – C) Cells have been gated on CD3+CD8− events. The FACS plots depict the IFN? response among CD3+CD8− T cells of representative patients from groups A, P, and N respectively. (D) Summary of data points from all patient samples analysed: group A (n = 37), group P (n = 44), and group N (n = 40). NS: not significant. (TIF) [file pone.0039199.s002.tif]
